# Effectiveness of Pain Neuroscience Education in Reducing Pain, Disability, Kinesiophobia, and Catastrophizing in Patients with Chronic Low Back Pain: A Systematic Review and Meta-Analysis

**DOI:** 10.3390/medsci13040290

**Published:** 2025-11-27

**Authors:** Luisa Medina-Viedma, Irene Cortés-Pérez, Esteban Obrero-Gaitán, María Catalina Osuna-Pérez, Ángeles Díaz-Fernández, María del Carmen López-Ruiz, Noelia Zagalaz-Anula

**Affiliations:** Department of Health Sciences, University of Jaen, Campus Las Lagunillas s/n, 23071 Jaen, Spain; lmv00031@red.ujaen.es (L.M.-V.); icortes@ujaen.es (I.C.-P.); mcosuna@ujaen.es (M.C.O.-P.); andiaz@ujaen.es (Á.D.-F.); mlruiz@ujaen.es (M.d.C.L.-R.); nzagalaz@ujaen.es (N.Z.-A.)

**Keywords:** chronic pain, low back pain, cognitive neuroscience, kinesiophobia, catastrophizing

## Abstract

**Background and objectives**: Pain neuroscience education (PNE) is a therapeutic strategy aimed at reconceptualizing pain in patients with chronic low back pain (CLBP). This systematic review with a meta-analysis (SRMA) aimed to assess the effectiveness of PNE in reducing pain, disability, kinesiophobia, and catastrophizing in patients with CLBP at the end of the intervention, and at 1 and 3 months of follow-up. **Materials and Methods**: Following PRISMA guidelines, an SRMA was conducted after searching in PubMed Medline, Scopus, Web of Science, and PEDro databases from inception up to June 2025. The inclusion criteria agreed with the PICOS tool: population (patients with CLBP), intervention (PNE), comparator (physiotherapy or non-intervention), outcomes (pain, disability, kinesiophobia, and catastrophizing), and study design (randomized controlled trials (RCTs) and pilot RCTs). The PEDro scale was used to assess the methodological quality and risk of bias of the RCTs included. The pooled effect was assessed using the Cohen standardized mean difference (SMD) and its 95% confidence interval (95% CI) in a random-effects model. **Results**: Fifteen RCTs, including data from 810 patients (43.7 ± 5.2 years; 61% female) with CLBP were included. The mean methodological quality of the RCTs included was good (6.8 ± 1.1 on the PEDro scale). Selection, performance, and detection were the most important biases identified. Our meta-analysis demonstrated, at the end of the intervention, and at 1 and 3 months of follow-up, respectively, that PNE is effective in reducing pain intensity (SMD = −0.65, *p* = 0.005; SMD = −1.1, *p* < 0.001; SMD = −1; *p* < 0.001), disability (SMD = −0.6, *p* = 0.009; SMD = −0.78, *p* = 0.002; SMD = −0.84; *p* = 0.004), and kinesiophobia (SMD = −1.12, *p* < 0.001; SMD = −1.51, *p* < 0.001; SMD = −1.57; *p* = 0.001). In reducing catastrophizing, PNE was largely effective at the end of intervention (SMD = −0.9, *p* = 0.016) and at 1 month of follow-up (SMD = −1.36, *p* = 0.007). **Conclusions**: Our findings demonstrate that PNE is an effective therapeutic approach for the management of CLBP, reducing pain, disability, kinesiophobia, and catastrophizing in patients with CLBP.

## 1. Introduction

Low back pain is defined as pain located below the costal margins and above the gluteal folds. On the one hand, it can be classified according to the origin of the symptoms as non-specific (not due to any specific known pathology, such as mechanical low back pain) or specific (the cause is due to a specific known pathology such as a tumor, fracture, infection, etc.), and on the other hand, it is classified according to the duration of the symptoms as acute (duration of less than 6 weeks), subacute (between 6 and 12 weeks), or chronic (more than 12 weeks) [1]. In terms of its assessment, the most recommendable is that which is within a biopsychosocial framework; therefore, different factors beyond the physical ones must be examined, such as genetic, patho-anatomical, neurophysiological characteristics of pain, psychological, and social factors [2].

Chronic low back pain (CLBP) is a pathological condition that concerns the entire healthcare workforce because it is the leading cause of disability worldwide [3]. Moreover, its persistence is characterized by the recurrence of symptoms, in most patients, at least once a year, which increases the number of medical visits, sick leave, and high medical expenses [4].

Currently, the most recommended physiotherapy approaches for CLBP management are exercise, manual therapy, and/or other direct techniques, as well as patient education [5]. Within exercise there are different modalities that help to improve the symptoms of CLBP, such as general training, strengthening, and increasing muscular endurance of the trunk, specific activation of the spinal muscles, aerobic and aquatic exercises, and multimodal exercise. In addition, motor control and mobility exercises for the trunk can also be included. When applying for patient education, pain neuroscience education (PNE) is one of the most recommended options, especially when combined with interventions such as exercise or manual therapy, to reduce both pain and disability [5]. Other alternatives are standard or general education in combination with other interventions and active treatments such as Pilates or yoga [5,6,7].

PNE is an educational method, within a biopsychosocial model, for patients with painful musculoskeletal conditions. It aims to reconceptualize pain by making patients understand the complex neurophysiological, neurobiological, sociological, and physical mechanisms behind it. Metaphors and analogies are their main tools to explain the neurophysiology of pain and the other multidimensional factors that influence the painful experience [8]. The objectives and content of PNE are grouped in a single book widely known by those interested in implementing PNE in their treatments [9]; this is the book *Explain Pain* by Butler, D.S. et al. [10]. Another avenue or concept through which this type of treatment is often applied is the guide *Therapeutic Neuroscience Education* by Louw and Puentedura [11]. The beginnings of this “methodology” date back to the early 2000s; therefore, there have been certain advances both in the field of PNE research and in society, forcing a reconceptualization of the initial concept, thus giving rise to the term ‘pain science education’ (PSE), where updated educational strategies are implemented [9]. There is no consensus on how to apply the treatment; this can be through group explanations or individually, using presentations, books, or brochures that complement the explanations, all being totally valid.

Pain, disability, kinesiophobia, and catastrophic attitudes towards pain are all characteristics that can be observed in CLBP patients. Pain, according to the current definition of the International Association for the Study of Pain (IASP), is “an unpleasant sensory and emotional experience associated with, or resembling that associated with, actual or potential tissue damage” [12]. On the one hand, the International Classification of Functioning, Disability, and Health (ICF) [13] explains disability as a concept that encompasses deficits, activity limitations, and restrictions in a person’s participation that are influenced by both internal and external factors. On the other hand, kinesiophobia is the excessive, irrational, and debilitating fear of physical movement and activity, caused by a feeling of vulnerability to injury or re-injury; it affects 51–72% of chronic pain patients and accentuates both disability and hypervigilance, triggering increased pain sensation [14]. Finally, catastrophizing pain is a persistent negative cognitive and emotional response to painful sensations, such that the sufferer tends to exaggerate or magnify the severity of the pain or the threat value [15].

CLBP is a growing global health concern with major socioeconomic implications [3]. Traditional biomechanical approaches often overlook the cognitive and emotional dimensions that sustain pain chronicity. This underscores the urgent need for biopsychosocial interventions such as PNE. Therefore, it is considered imperative to conduct the present systematic review to address this need by consolidating and critically appraising the current evidence, thereby informing clinicians and guideline developers seeking to integrate PNE into the management of CLBP.

To date, there are three systematic reviews [4,16,17] that evaluated the effects of PNE applied to patients with CLBP. These previously published reviews have some limitations that may reduce the impact of their results, such as the low number of studies included in these reviews, the small sample size of some of the included studies, or the language restrictions in the search strategy for the reviewed studies. The limitations discussed in these previous reviews, along with the publication of several recent randomized controlled trials (RCTs), make it necessary to conduct a new review to establish more stable conclusions with a higher level of evidence. Therefore, the objective of this systematic review with a meta-analysis (SRMA) was to retrieve all scientific evidence currently available on the application of PNE in patients with CLBP to assess the effectiveness of PNE in reducing pain, disability, kinesiophobia, and catastrophizing in patients with CLBP at three time points: at the end of the intervention, and at 1 and 3 months of follow-up.

## 2. Materials and Methods

### 2.1. Protocol Design

This SRMA was conducted following the Preferred Reporting Items for Systematic Reviews and Meta-Analyses (PRISMA) 2020 guidelines [18] and the Cochrane Handbook for Systematic Reviews of Interventions [19]. The protocol of this SRMA was previously registered in the PROSPERO database: CRD420251167278.

### 2.2. Search Strategy and Data Sources

The literature search was carried out by two authors independently in PubMed Medline, Scopus, Web of Science (WOS), and PEDro (Physiotherapy Evidence Database) databases from inception up to June 2025. Additionally, the authors searched the list of references of RCTs included and in abstracts and proceedings. The PICOS [20] tool was adopted: population (P): patients with CLBP; intervention (I): PNE; comparison (C): other physiotherapy treatments or usual care; outcomes (O): pain, disability, kinesiophobia, and catastrophizing; and study design (S): RCTs and/or pilot RCTs. The keywords used in the search strategy, together with their synonyms, were “pain neuroscience education”, “chronic low back pain”, “disability”, “chronic pain”, “kinesiophobia”, and “catastrophizing”. The keywords and their synonyms were combined with the Boolean operators ‘AND’ or ‘OR’. Table 1 shows the specific search strategies for each database.

### 2.3. Study Selection: Inclusion and Exclusion Criteria

Two authors, independently, assessed the studies retrieved from literature search by title/abstract. A third author was in charge to resolve discrepancies. The studies included in this SRMA had to meet the PICOS criteria: (1) RCTs and pilot RCTs; (2) including patients with CLBP; (3) in which one group received PNE and the comparison group other therapy different to PNE; (4) and that provided quantitative data susceptible to be included in the meta-analysis of the outcomes of interest (pain intensity, disability, kinesiophobia, and catastrophizing). As exclusion criteria, we excluded studies that did not provide data from patients with exclusive CLBP.

### 2.4. Data Extraction

Two authors independently extracted the following data into a Microsoft Excel data-collection sheet, and a third author was consulted to resolve disagreements. We extracted the following data: (1) general characteristics of the study (authorship and publication date, study design, total sample size); (2) characteristics of the intervention and control groups (number of participants, mean age, time with CLBP in months, intervention and duration of the intervention in weeks, number of sessions per week, and duration of each session in minutes); (3) data related to the outcomes (variables assessed and measurement tools used, and quantitative data necessary to perform the meta-analysis [mean and standard deviation post-intervention]); and (4) evaluation time sequence (right at the end of the therapy or follow-up period).

### 2.5. Assessment of the Methodological Quality, Risk of Bias

Two authors were in charge of assessing the risk of bias and methodological quality of the studies included in the review, using the PEDro scale [21]. Disagreements were solved by a third author. It is a widely used scale and a valid tool to assess the methodological quality of studies in the field of physiotherapy [22]. This scale consists of 11 criteria that are answered with “yes” if the criterion is fulfilled (adds 1 point) or “no” if it is not fulfilled (0 points). In this review, RCTs can show excellent (10−9 points), good (8−6 points), moderate (5−4 points), or poor methodological quality (3−0 points). A lower risk of bias could be correlated with higher scores. Although the PEDro scale and the Cochrane Risk of Bias tool are structured differently (one assesses items independently), they both measure the same types of biases. Selection, performance, and detection biases can be addressed if the items 2–3, 5–6, and 7 on the PEDro scale, respectively, are not met.

### 2.6. Statistical Analysis

The meta-analysis was conducted by two authors using Comprehensive Meta-Analysis version 4 (Biostat, Englewood, NY, USA) [23]. Firstly, for the first meta-analysis, Cohen’s standardized mean difference (SMD) with its 95% confidence interval (95% CI) was used as a pooled effect measure for continuous data in a random-effects model [24]. The effect size was interpreted according to Kinney et al. (2020) for rehabilitation studies [25]. Each meta-analysis was graphically displayed in a forest plot [26]. The funnel plot, *p*-value for the Egger test, and the trim-and-fill estimation were used to determine the risk of publication bias [27,28,29]. Inconsistency or heterogeneity was calculated with the degree of inconsistency of Higgins, the χ-square test, and its *p*-value [30]. According to this, heterogeneity can be large (I^2^ > 50%), medium (I^2^ > 50–25%), low (I^2^ 25–5%), or null (I^2^ < 5%) [31]. Finally, as an additional analysis, a sensitivity analysis using the leave-one-out method was conducted to assess the contribution of each study to the pooled effect.

## 3. Results

### 3.1. Study Selection and Flow Diagram

**Figure 1:**
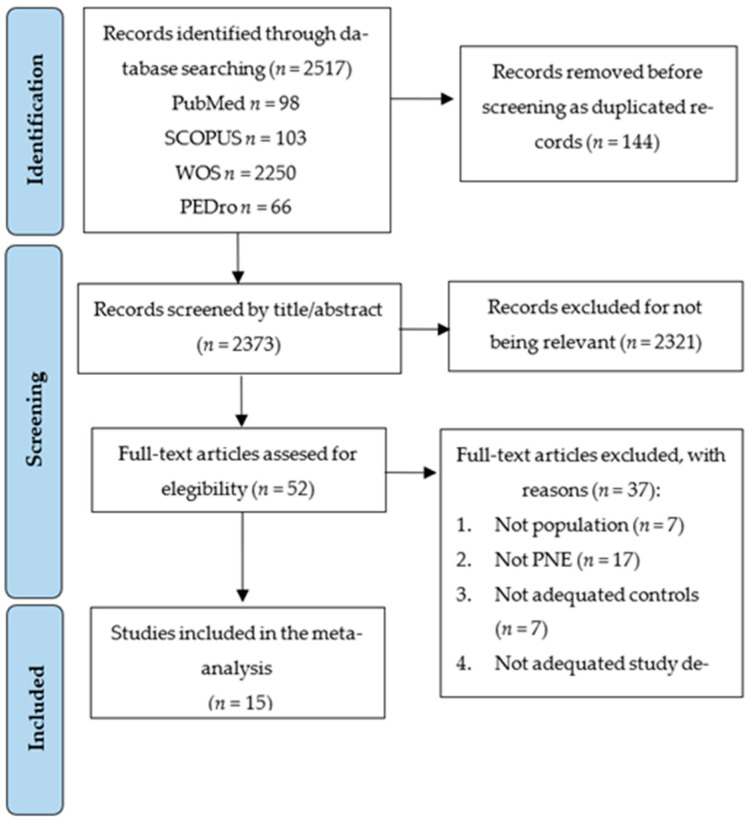
PRISMA flow chart for the study selection process.

The PRISMA 2020 flow diagram (Figure 1) displays the study selection process. A total of 2517 studies were retrieved from the initial literature search, of which 144 were removed as duplicated. Of the remaining 2373 studies, 2321 were excluded for not being relevant by title/abstract. Fifty-two records were assessed for eligibility, excluding thirty-seven studies that did not meet the inclusion criteria (reasons in Figure 1). Finally, 15 RCTs were included in this SRMA [32,33,34,35,36,37,38,39,40,41,42,43,44,45,46].

### 3.2. Characteristics of the Studies Included in the Review

The RCTs included were conducted in Turkey, Spain, USA, Brazil, India, South Korea, Portugal, and Australia between 2004 and 2024. These RCTs provided data from 810 patients with CLBP (43.7 ± 5.2 years, approximately 61% women), of whom 407 belonged to the experimental intervention group (PNE), and 403 received the control interventions. In the experimental group, the intervention consisted of the application of PNE in combination with exercise or different physiotherapy techniques such as manual therapy, soft tissue mobilization, or dry needling, except in two RCTs [41,46], in which PNE was applied in isolation. On the other hand, in the control group, the interventions included different physiotherapy techniques consisting of the same exercise and physical therapy techniques as in the experimental group, except in the study of Ünal, M. et al. (2020) [41]. The RCTs included provided data of three time points of assessment since the end of the intervention: at the end of the intervention (T1) and at 1 (T2) and 3 months since the end of intervention (T3). Table 2 lists the characteristics of the RCTs included in this SRMA.

### 3.3. Methodological Quality and Risk of Bias Assessment of the Studies Included in the Review

The methodological quality mean of the RCTs included was good, exhibiting a mean score of 6.8 ± 1.1 on the PEDro scale. Thirteen RCTs (87% of all) reported good methodological quality (8−6 points), and in two RCTs (13%), the methodological quality was moderate (5 points) [39,41]. The PEDro database provided the risk of bias for 10 studies [32,35,36,37,38,44,45] and the rest were assessed manually. According to the risk of bias, we identified the following biases in the RCTs included. A risk of selection bias was reported by five RCTs due to an inadequate concealed allocation (item 3). A risk of performance bias was present in all RCTs due to the impossibility of blinding participants (except Moseley, GL et al., 2004, [46]) and therapists. Finally, detection bias, since evaluators were not blinded, was present in six RCTs. Table 3 highlights the PEDro score and biases of each RCT included.

### 3.4. Quantitative Synthesis: Meta-Analysis

#### 3.4.1. Pain

**Figure 2:**
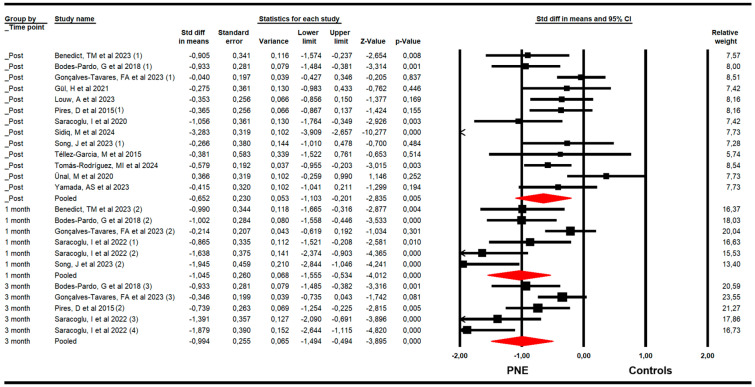
Forest plot of the effectiveness of PNE in reducing pain at the end of the intervention, and at 1 and 3 months of follow-up [32,33,34,35,36,37,38,39,40,41,42,43,44,45].

For pain, RCTs included combining data from the Numeric Pain Rating Scale (NPRS), Visual Analogue Scale (VAS for pain), and McGill Pain Questionnaire (MGPQ). Our findings demonstrated that PNE is largely effective in reducing pain just to the end of the intervention (k = 13; n = 712 participants; n_s_ = 54.76 per comparison; SMD = −0.65; 95% CI −1.1 to −0.2; *p* = 0.005; I^2^ = 11.1%; Q = 13.5; df = 12; *p* = 0.34), and at one (k = 6; n = 293 participants; n_s_ = 48.83 per comparison; SMD = −1.05; 95% CI −1.56 to −0.53; *p* < 0.001; I^2^ = 0%; Q = 4.5; df = 5; *p* = 0.48) and three months of follow-up (k = 5; n = 298 participants; n_s_ = 59.6 per comparison; SMD = −1; 95% CI −1.5 to −0.5; *p* < 0.001; I^2^ = 4.6; Q = 4.2; df = 4; *p* = 0.38) (Figure 2). The sensitivity analysis did not report substantial variation in any meta-analysis. The risk of publication bias was not present in any meta-analysis. The risk of publication bias only was suggested (Egger *p* = 0.26) in the meta-analysis of the post-intervention assessment, in which the variation in effect size after the trim-and-fill estimation (adjusted SMD = −1) was 35% with respect to the original pooled effect (Supplementary Figure S1).

#### 3.4.2. Disability

**Figure 3:**
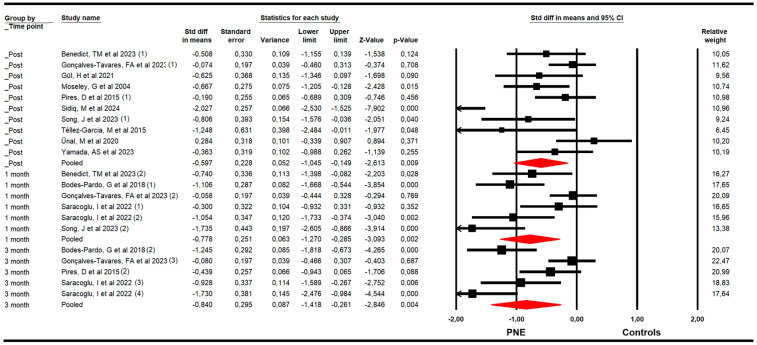
Forest plot of the effectiveness of PNE in reducing disability at the end of the intervention, and at 1 and 3 months of follow-up [32,33,34,35,36,37,38,39,40,41,42,44,45].

The effectiveness of PNE in reducing disability was assessed with data from the Roland Morris Disability Questionnaire (RMDQ) and the Oswestry Disability Index (ODI). The meta-analysis revealed a large effect size of PNE on reducing disability just to the end of the intervention (k = 10; n = 502 participants; n_s_ = 50.2 per comparison; SMD = −0.6; 95% CI −1.11 to −0.15; *p* = 0.009; I^2^ = 0%; Q = 7.5; df = 9; *p* = 0.58), and at 1 (k = 6; n = 302 participants; n_s_ = 50.3 per comparison; SMD = −0.78; 95% CI −1.27 to −0.29; *p* = 0.002; I^2^ = 0%; Q = 4.7; df = 5; *p* = 0.45) and 3 months since the end of the intervention (k = 5; n = 298 participants; n_s_= 59.6 per comparison; SMD = −0.84; 95% CI −1.42 to −0.26; *p* = 0.004; I^2^ = 0%; Q = 3.9; df = 4; *p* = 0.42) (Figure 3). The leave-one-out method reported an equal contribution of studies in each pooled effect. The risk of publication bias was advertised at the 3-month assessment’s meta-analysis. The funnel plot was asymmetric (Egger *p* = 0.02) and the trim-and-fill calculation reported an adjusted effect size (adjusted SMD = −0.44) 90% lower than the original pooled effect considering the risk of publication bias (Supplementary Figure S2).

#### 3.4.3. Kinesiophobia

**Figure 4:**
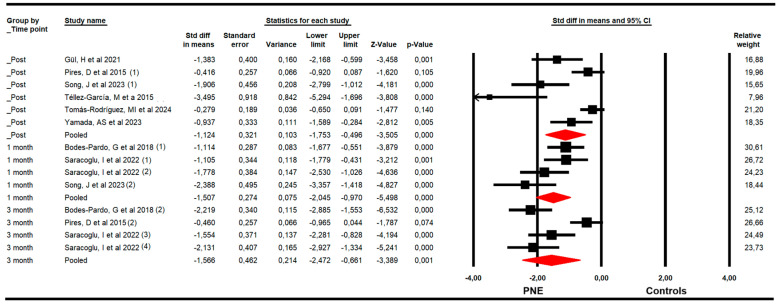
Forest plot of the effectiveness of PNE in reducing kinesiophobia at the end of the intervention, and at 1 and 3 months of follow-up [34,35,38,39,42,44,45].

The effectiveness of PNE in decreasing kinesiophobia was evaluated with data from the Tampa Kinesiophobia Scale (TSK). The meta-analysis showed a large effect size of PNE on decreasing kinesiophobia just to the end of the intervention (k = 6; n = 276 participants; n_s_ = 46 per comparison; SMD = −1.12; 95% CI −1.8 to −0.5; *p* < 0.001; I^2^ = 36.9%; Q = 7.9; df = 5; *p* = 0.17), and at 1 (k = 4; n = 161 participants; n_s_ = 40.3 per comparison; SMD = −1.51; 95% CI −2.1 to −0.97; *p* < 0.001; I^2^ = 10.2%; Q = 3.3; df = 3; *p* = 0.35) and 3 months since the end of the intervention (k = 4; n = 194 participants; n_s_ = 48.5 per comparison; SMD = −1.57; 95% CI −2.47 to −0.66; *p* = 0.001; I^2^ = 0%; Q = 2.4; df = 3; *p* = 0.5) (Figure 4). The risk of publication bias was advertised in the meta-analysis of the post-intervention assessment. The funnel plot was asymmetric (Egger *p* < 0.001), and the trim-and-fill calculation reported an adjusted effect size (adjusted SMD = −0.70) 84% lower than the original pooled effect considering the risk of publication bias (Supplementary Figure S3).

#### 3.4.4. Catastrophizing

**Figure 5:**
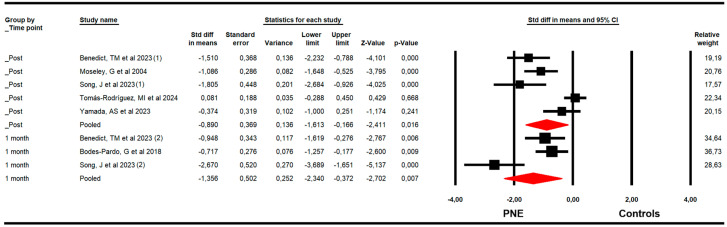
Forest plot of the effectiveness of PNE in reducing catastrophizing at the end of the intervention, and at 1 and 3 months of follow-up [32,34,35,37,42,46].

The effectiveness of PNE on catastrophizing was assessed with data from the Pain Catastrophizing Scale (PCS). The meta-analysis showed a large effect size of PNE on decreasing catastrophizing just to the end of the intervention (k = 5; n = 275 participants; n_s_ = 55 per comparison; SMD = −0.9; 95% CI −1.61 to −0.17; *p* = 0.016; I^2^ = 0%; Q = 3.6; df = 4; *p* = 0.46), and at 1 (k = 3; n = 122 participants; n_s_ = 40.7 per comparison; SMD = −1.36; 95% CI −2.34 to −0.37; *p* = 0.007; I^2^ = 28.3%; Q = 2.8; df = 2; *p* = 0.25) month after follow-up (Figure 5). The leave-one-out method reported an equal contribution of studies in each pooled effect. No risk of publication bias was reported in any meta-analysis.

## 4. Discussion

CLBP is ranked as the most prevalent chronic condition worldwide and constitutes one of the most important public health problems today [47]. Furthermore, it is a complex pathology where physical, social, and psychological factors interact with each other, and, therefore, treating it from a biopsychosocial perspective is the most appropriate option [48]. PNE is one of the interventions that should be applied within the biopsychosocial treatment of CLBP. Therefore, the objective of the present SRMA was to collect all available evidence to assess the effectiveness of PNE for reducing pain, disability, kinesiophobia, and catastrophizing attitudes in patients with CLBP at the end of the intervention and after 1 and 3 months since the end of the intervention.

The use of PNE for CLBP has been previously assessed. Currently, three systematic reviews prior to this one have dealt with the same topic, two including a meta-analysis [4,16] and one without a meta-analysis [17]. Wood, L. et al., (2019) [16], which included eight RCTs, concluded that the use of PNE as an adjunct to physiotherapy treatments reduced both disability and pain in the short term. Ma, X. et al. (2024) also corroborated short-term improvements in kinesiophobia and catastrophizing [4]. The most recent review, by Iken, A. et al. (2024) [17], included six RCTs and aimed to evaluate the impact of PNE duration on kinesiophobia and pain catastrophizing. Based on the chosen RCTs, their result showed no significant correlation between the duration of education sessions and these variables.

Following the purpose of this SRMA, after a literature search and application of inclusion and exclusion criteria, 15 RCTs were included in the meta-analysis to analyze the effects of PNE on the aforementioned variables. First, about pain intensity, our findings showed that PNE is effective in reducing pain at the end of the intervention, and at 1 and 3 months of follow-up. Previously, MA, X et al. also found statistically significant results in short-term pain improvement after treatment of CLBP with PNE [4]. These authors considered short-term outcomes to be those obtained ≤12 weeks after randomization. They indicated that if two or more short-term follow-up assessments were performed within the same study, the one closest to the time of data extraction was used. Therefore, our meta-analysis included more information by differentiating, within the short-term outcomes, the effect at the end of treatment, at one month, and at three months of follow-up. On the other hand, a previous meta-analysis presented by Wood L et al. also analyzed the short-term pain variable, without making the temporal distinction (post-treatment, and at 1 and 3 months) that we made in the present SRMA, and with a smaller number of included studies (of the eight articles included in the meta-analysis, only six analyzed the pain variable) [16]. These authors found that PNE combined with physiotherapy or exercise produced significant short-term improvements.

Continuing with disability, in our meta-analysis, we found that PNE treatment produces statistically significant reductions in disability in patients with CLBP at the end of the intervention, at 1 month and 3 months of follow-up. Ma, X et al. also found significant and clinically relevant improvements in disability [4]. Wood L et al. found that disability improved in the short term when combined with physiotherapy and exercise, although only disability achieves clinical relevance [16].

Regarding the psychosocial variables evaluated in this SRMA (kinesiophobia and catastrophizing), we found significant reduction in kinesiophobia at the end of the intervention, and at 1 month and 3 months of follow-up, and also significant results in favor of PNE treatment for catastrophizing in the immediate follow-up and at one month after the intervention (there were no data to analyze the effect of PNE on catastrophizing at 3 months). These two variables were only previously analyzed by Ma, X et al., who also found significant results for both variables in the short term [4].

Therefore, after all the above, we can say that PNE could be a good tool to be considered for use in conjunction with other interventions [5,49] in patients with CLBP, even in those with high levels of catastrophizing and kinesiophobia. Some hypotheses that may explain the statistically significant improvements in the different variables are that thanks to PNE prior to treatments such as therapeutic exercise, (1) changes can be generated in the “pain matrix” at the central level, thanks to the replacement of old and maladaptive pain memories related to movement [50]; (2) changes in pain cognition and pain self-control, produced by PNE, can directly influence pain intensity, and this effect can be prolonged over time with the repetition of several sessions to consolidate the information [51]; and (3) greater knowledge of pain is associated with less kinesiophobia [52], while the latter is also related to the level of disability [53].

In terms of clinical practice, PNE appears to be an effective intervention for patients with CLBP, as current evidence indicates improvements in pain-related outcomes, particularly in reducing kinesiophobia and catastrophizing. Clinicians can immediately incorporate PNE into treatment plans by integrating short, structured educational sessions that explain the neurophysiology of pain using metaphors, stories, and patient-centered discussions. These sessions can be delivered individually or in groups and may be supported by resources such as the “Explain Pain” [10] or “Therapeutic Neuroscience Education” [11] guidelines. Regarding clinical implementation, previous studies suggest that the minimum effective “dose” of PNE depends on the outcome targeted. Approximately 200 min of total PNE [54] exposure appears to be the threshold for significant improvements in pain intensity when combined with exercise, while 150 min [54] may be sufficient to improve disability outcomes, and session durations exceeding 100 min may be associated with a greater reduction in kinesiophobia and catastrophizing [17]. However, these data should be interpreted with caution, as they come from isolated clinical RCTs or reviews without meta-analysis. Therefore, further studies are needed to corroborate this information. From a practical standpoint, these doses can be achieved through two to four sessions of 30–60 min each, depending on the setting and patient needs. PNE can be delivered through interactive sessions—either individually or in small groups—using visual support and metaphors to promote reconceptualization of pain [11]. Moreover, integrating PNE before or alongside active interventions such as exercise or manual therapy may further enhance its benefits. Nevertheless, as there is still no fully established dose–response relationship, future studies should aim to determine the optimal duration, frequency, and mode of delivery to facilitate standardization and comparability among clinical trials.

Finally, this SRMA presents limitations that must be highlighted. Firstly, although a literature search was conducted according to gold standard guidelines for SRMA, Chinese and Japanese databases were not searched. Second, the low number of RCTs included, especially in some meta-analyses, and overall, the low number of participants, can reduce the generalization and precision of our findings. Moreover, it is important to consider the presence of selection, performance, and detection biases in the studies included that can influence the findings, since these can underestimate or overestimate the true effect of PNE [55,56]. Moreover, it is important to highlight the risk of publication bias suggested in some meta-analyses. However, trim-and-fill estimation reported the adjusted effect size by taking into account the risk of publication bias, demonstrating if publication bias underestimated or overestimated the findings. Other limitations were related to the heterogeneity in PNE protocols and comparators of the studies included, as it is difficult to obtain homogeneous findings. Finally, the effect of PNE at 6 or 12 months could not be assessed due to the absence of data in the RCTs included. Therefore, further studies investigating the effectiveness of PNE in patients with CLBP of higher methodological quality and with larger sample sizes and longer-lasting interventions are recommended. However, it is noteworthy that, in relation to previous reviews [4,16,17], the present review updates all the evidence available today and excludes studies where other treatments overshadow the effects of PNE.

## 5. Conclusions

This SRMA highlights that PNE is an effective therapeutic strategy for patients with CLBP, demonstrating its effectiveness for reducing pain, disability, kinesiophobia, and catastrophizing. For pain, disability, and kinesiophobia, PNE demonstrated superior effectiveness compared to control interventions immediately following the intervention. Crucially, this beneficial effect was maintained at the 1- and 3-month follow-ups. Regarding catastrophizing, the effectiveness of PNE was assessed and confirmed at the end of the intervention and at the 1-month follow-up. However, these findings should be interpreted with caution due to the variability observed in the PNE and control interventions across the included studies. Therefore, future studies must perform homogeneous PNE protocols assessing its effect at 6- and 12-month follow-ups with the aim to demonstrate its long-term utility in routine clinical practice.

## Figures and Tables

**Table 1:** Search strategy for each database.

Databases	Search Strategy
PubMed Medline	(pain education [tiab] OR pain neuroscience education [tiab] OR PNE [tiab] OR neuroscience education [tiab] OR therapeutic neuroscience education [tiab] OR pain science education [tiab] OR neuroscience pain education [tiab] OR pain neurophysiology education [tiab]) AND (chronic low back pain [tiab] OR recurrent low back pain [tiab]) AND (chronic pain [mh] OR chronic pain [tiab] OR disability [tiab] OR kinesiophobia [mh] OR kinesiophobia [tiab] OR fear of movement [tiab] OR kinesophobia [tiab] OR movement fear [tiab] OR exercise phobia [tiab] OR catastrophizing [mh] OR catastrophizing [tiab] OR catastrophic thinking [tiab] OR catastrophizing [tiab])
Scopus	(TITLE-ABS-KEY (“pain education” OR “pain neuroscience education” OR “PNE” OR “neuroscience education” OR “therapeutic neuroscience education” OR “pain science education” OR “pain neurophysiology education”) AND TITLE-ABS-KEY (“chronic low back pain” OR “recurrent low back pain”) AND TITLE-ABS-KEY (“chronic pain” OR “disability” OR “kinesiophobia” OR “fear of movement” OR “kinesophobia” OR “movement fear” OR “exercise phobia” OR “catastrophizing” OR “catastrophic thinking” OR “catastrophizing”))
Web of Science	TOPIC: (*pain education* OR *pain neuroscience education* OR *PNE* OR *neuroscience education* OR *therapeutic neuroscience education* OR *pain science education* OR *pain neurophysiology education*) AND TOPIC: (*chronic low back pain* OR *recurrent low back pain*) AND TOPIC: (*chronic pain* OR *disability* OR *kinesiophobia* OR *fear of movement* OR *kinesophobia* OR *movement fear* OR *exercise phobia* OR *catastrophizing* OR *catastrophic thinking* OR *catastrophizing*)
PEDro	Pain neuroscience education AND chronic low back pain
	Pain science education AND chronic low back pain
	Therapeutic neuroscience education AND chronic low back pain

**Table 2:** Characteristics of the studies included in the review.

			Experimental Group							Control Group							Outcomes		
			Sample Characteristics			Intervention Characteristics				Sample Characteristics			Intervention Characteristics						
Authorship and Date	Country	N	N_e_	Age	Evol. Months	Type	Weeks	Ses/Week	Min/Ses	N_c_	Age	Evol. Months	Type	Weeks	Ses/Week	Min/Ses	Variable	Test	Time-Point Assessment
Benedict, TM. et al., 2024 [32]	USA	38	18	37.2 ± 9.7	64.3 ± 61.3	PNE + Exercise	4	1	PNE: 30 Exercise: 15 Total: 60	20	41.7 ± 11	102.9 ± 95.1	TE + Exercise	4	1	ET: 30 Exercise: 15 Total: 60	Pain	NPRS	T1 T2
																	Disability	RMDQ	
																	Catastrophizing	PCS	
Sidiq, M. et al., 2024 [33]	India	92	46	42 ± 8.2	7.8 ± 1.3	PNE + PT	6	1	40	46	42.5 ± 13.3	7.4 ± 1.2	PT	6	1	40	Pain	VAS	T1
																	Disability	RMDQ	
Tomás-Rodríguez MI. et al., 2024 [34]	Spain	113	56	53.9 ± 14.1	>3	PNE + BS + Exercise	5	PNE: 1 extra Ses in the 1st week. BS: 1	PNE: 60 BS: 60	57	57.5 ± 13	>3	BS + Exercise	5	BS: 1	BS: 60	Pain	NPRS	T2
																	Catastrophizing	PCS	
																	Kinesiophobia	TSK	
Song, J. et al., 2023 [35]	South Korea	28	14	45.6 ± 12.3	7.95 ± 3.15	PNE + STM	4	PNE: only 2 Ses in total. STM: 2	PNE: 30–50 STM: 40	14	45.2 ± 12.3	9.48 ± 2.88	STM	4	2	STM: 40	Pain	NPRS	T1 T2
																	Disability	RMDQ	
																	Catastrophizing	PCS	
																	Kinesiophobia	TSK	
Gonçalves-Tavares, FA. et al., 2023 [36]	Brazil	104	52	38.8 ± 11.6	>3	PNE + MT	4	PNE: only 2 Ses in total in the 1st week. MT: 2	PNE: 40 MT: -	52	41.6 ± 9.7	>3	MT	4	2	-	Pain	NPRS	T1 T2 T3
																	Disability	ODI	
Yamada, AS. et al., 2023 [37]	Brazil	40	20	44.5 ± 17.5	- 3–12 months: 30% - 13–60 months: 30% - >60 months: 40%	PNE + PT	6	PNE: 3 extra Ses PT: 2	PNE: 50 PT: 50	20	50.1 ± 14.1	- 3–12 months: 35% - 13–60 months: 25% - >60 months: 40%	PT	6	2	50	Pain	NPRS	T1
																	Disability	RMDQ	
																	Catastrophizing	PCS	
																	Kinesiophobia	TSK	
Saracoglu, I. et al., 2022 [38]	Turkey	39	20	39.7 ± 13.7	28.14 ± 16.62	PNE + MT + Exercise for home	4	PNE: 1 MT: 2 Exercise for home: 7	PNE: 40–45 MT: 30	19	41.4 ± 12.7	34.6 ± 15.37	MT + Exercise for home	4	MT: 2 Exercise for home: 7	MT: 30	Pain	NPRS	T2 T3
																	Disability	ODI	
																	Kinesiophobia	TSK	
Gül, H. et al., 2021 [39]	Turkey	31	16	42.1 ± 10.1	≥3	TNE + PT	3	TNE: 2 PT: 5	TNE: 40 PT: 50	15	42.5 ± 12	≥3 months	PT	3	5	40	Pain	VAS	T1
																	Disability	RMDQ	
																	Kinesiophobia	TSK-11	
Saracoglu, I. et al., 2020 [40]	Turkey	35	17	39.8 ± 14.2	27.5 ± 14.94	PNE + MT	4	PNE: 1 MT: 2	PNE: 45–50 MT: 40–45	18	38.9 ± 12.5	36.94 ± 15.2	TE + MT	4	TE: only 1 Ses in total MT: 2	TE: 45–50 TM: 40–45	Pain	NPRS	T1
Ünal, M. et al., 2020 [41]	Turkey	40	20	42.6 ± 7.9	14.15 ± 4.13	PNE	8	2	40	20	41.3 ± 9.1	14.35 ± 3.76	MI	8	2	40	Pain	MPQ	T1
																	Disability	RMDQ	
Bodes-Pardo, G. et al., 2018 [42]	Spain	56	28	44.9 ± 9.6	≥6	PNE + TEx + Exercise for home	12	PNE: only 2 Ses in total TEx: only 2 Ses in total. Exercise for home: 7	PNE: 30–50 TEx: 30–50	28	49.2 ± 10.5	≥6	TEx + Exercise for home	12	TEx: only 2 Ses in total. Exercise for home: 7	TEx: 30–50	Pain	NPRS	T1 (only for NPRS) T2 T3
																	Disability	RMDQ	
																	Catastrophizing	PCS	
																	Kinesiophobia	TSK	
Louw, A. et al., 2017 [43]	USA	62	33	-	-	NE + MT	1	NE: 1 MT: 1	NE: 5 MT: 10	29	-	-	BE + MT	1	BE: 1 MT: 1	BE: 5 MT: 10	Pain	NPRS	T1
Pires, D. et al., 2015 [44]	Portugal	62	30	50.9 ± 6.2	- 3–24 months: 20% - >24 months: 80%	PNE + AE	6	PNE: only 2 Ses in total in the 1st week AE: 2	PNE: 90 AE: 30–50	32	51 ± 6.3	- 3–24 months: 25% - >24 months: 75%	AE	6	2	30–50	Pain	VAS	T1 T3
																	Disability	QBPDS	
																	Kinesiophobia	TSK	
Téllez-García, M. et al., 2015 [45]	Spain	12	6	36 ± 5	17 ± 9	PNE + DN	3	PNE: only 2 Ses (one in the 2nd and the other in the 3rd week). DN: 1	PNE: 30 DN: -	6	37 ± 13	19 ± 8	DN	3	1	-	Pain	NPRS	T1
																	Disability	RMDQ	
																	Kinesiophobia	TSK	
Moseley, GL. et al., 2004 [46]	Australia	58	31	42 ± 10	29 ± 11	PNE	1 day	1	180	27	45 ± 6	30 ± 13	Education of the anatomy and physiology of the back	1 day	1	180	Disability	RMDQ	T1
																	Catastrophizing	PCS	

Abbreviations: N, total sample size; N_e_, experimental group sample size; Evol, evolution; Ses, sessions; Min, minutes; N_c_, control group sample size; T1, Post-intervention assessment; T2, 1 month of follow-up; T3, 3 months of follow-up; PNE, pain neuroscience education; PT, physiotherapy; BS, back school; STM, soft tissue mobilization; MT, manual therapy; TNE, therapeutic neuroscience education; TEx, therapeutic exercise; NE, neuroplasticity education; AE, aquatic exercise; DN, dry needling; TE, traditional education; MI, myofascial induction; BE, biomechanical education; NPRS, Numeric Pain Rating Scale; RMDQ, Roland Morris Disability Questionnaire; PCS, Pain Catastrophizing Scale; VAS, Visual Analogue Scale; NPRS, Numeric Pain Rating Scale; ODI, Oswestry Disability Index; TSK, Tampa Scale for Kinesiophobia; MPQ, McGill Pain Questionnaire; QBPDS, Quebec Back Pain Disability Scale.

**Table 3:** Methodological quality and risk of bias (PEDro scores) of the studies included in the review.

Study	Items											Total Score	Biases Identified
	i1	i2	i3	i4	i5	i6	i7	i8	i9	i10	i11		
Benedict, TM. et al., 2024 [32]	Y	Y	Y	Y	N	N	Y	N	N	Y	Y	6	Performance
Sidiq, M. et al., 2024 [33]	N	Y	Y	Y	N	N	Y	Y	Y	Y	Y	8	Performance
Tomás-Rodríguez MI. et al., 2024 [34]	N	Y	N	Y	N	N	N	Y	Y	Y	Y	6	Selection, performance, and detection
Song, J. et al., 2023 [35]	Y	Y	N	N	N	N	Y	Y	Y	Y	Y	6	Selection and performance
Tavares, FAG. et al., 2023 [36]	N	Y	Y	Y	N	N	N	Y	Y	Y	Y	7	Performance and detection
Yamada, AS. et al., 2023 [37]	Y	Y	Y	Y	N	N	Y	Y	Y	Y	Y	8	Performance
Saracoglu, I. et al., 2022 [38]	Y	Y	Y	Y	N	N	Y	Y	N	Y	Y	7	Performance
Gül, H. et al., 2021 [39]	Y	Y	N	Y	N	N	N	Y	N	Y	Y	5	Selection, performance, and detection
Saracoglu, I. et al., 2019 [40]	Y	Y	Y	Y	N	N	Y	Y	N	Y	Y	7	Performance
Ünal, M. et al., 2020 [41]	Y	Y	N	Y	N	N	N	Y	N	Y	Y	5	Selection, performance, and detection
Bodes-Pardo, G. et al., 2018 [42]	Y	Y	Y	Y	N	N	Y	Y	Y	Y	Y	8	Performance
Louw, A. et al., 2017 [43]	Y	Y	N	Y	N	N	N	Y	Y	Y	Y	6	Selection, performance, and detection
Pires, D. et al., 2015 [44]	Y	Y	Y	Y	N	N	Y	Y	Y	Y	Y	8	Performance
Téllez-García, M. et al., 2015 [45]	N	Y	Y	Y	N	N	Y	Y	N	Y	Y	7	Performance
Moseley, GL. et al., 2004 [46]	Y	Y	Y	Y	Y	N	N	Y	Y	Y	Y	8	Performance and detection

Abbreviations: i1: eligibility criteria; i2: random allocation; i3: concealed allocation; i4: baseline comparability; i5: blind subjects; i6: blind therapists; i7: blind assessors: i8. measures of at least one key outcome were obtained from more than 85% of the subjects initially allocated to groups; i9: intention-to-treat analysis; i10: between-group comparisons; i11: point estimates and variability; Y: yes; N: no. Notes: Eligibility criteria item does not contribute to total score.

## Data Availability

No new data were created or analyzed in this study.

## Supplementary Materials

The following supporting information can be downloaded at: https://www.mdpi.com/article/10.3390/medsci13040290/s1, Figure S1: Funnel plot for the effect of PNE in reducing pain at the end of the intervention; Figure S2: Funnel plot for the effect of PNE in reducing disability at 3 months; Figure S3: Funnel plot for the effect of PNE in reducing kinesiophobia at the end of the intervention; Table S1: PRISMA 2020 checklist.
